# Errata

**Published:** 1894-01

**Authors:** 


### ERRATA.

Page 12, line 2, read Libbertz' for Tibbertz'.

Page 12, line 32, read side for ide.

Page 13, line 24, read Libbertz' for Tibbertz'.

Page 14, line 1, read 101.8 degrees for 108.1 degrees.

Page 16, line 24, read breeding for weeding.

Page 41, after Author's name, read V. M. D. for V. D. M.

